# Achievement of pathological complete response with osimertinib for *EGFR*-mutated lung adenocarcinoma

**DOI:** 10.1186/s44215-023-00069-8

**Published:** 2023-09-21

**Authors:** Yasuhiro Fujita, Nobuyuki Take, Shinji Shinohara, Masataka Mori, Masatoshi Kanayama, Masaru Takenaka, Koji Kuroda, Shohei Shimajiri, Fumihiro Tanaka

**Affiliations:** 1 Second Department of Surgery, University of Occupational and Environmental Health, Kitakyushu, Japan; 2 Department of Pathology, School of Medicine, University of Occupational and Environmental Health, Kitakyushu, Japan

**Keywords:** Epidermal growth factor receptor, Activating mutation, Tyrosine-kinase inhibitor, Osimertinib, Salvage surgery, Pathological complete response

## Abstract

**Background:**

Tyrosine-kinase inhibitors (TKIs) of epidermal growth factor receptor (EGFR) usually provide a potent anti-tumor efficacy with robust radiographic response for non-small cell lung cancer (NSCLC) harboring activating mutations in the EGFR gene. However, first-generation EGFR-TKIs may provide only modest pathological response in the majority of *EGFR*-mutated NSCLC. Here, we present a case of *EGFR*-mutated adenocarcinoma in which a pathological complete response was revealed in histological specimens obtained during conversion surgery following systemic treatment using a third-generation EGFR-TKI.

**Case presentation:**

A 61-year-old Japanese man was admitted to our hospital for salvage surgery. Four months prior to the admission, he had been diagnosed with unresectable adenocarcinoma (clinical stage IIIA/T4N1MO) originating from the right lower lobe. Chest computed tomography had revealed a 3.4-cm tumor and enlarged hilar nodes that invaded into the left atrium through the lower pulmonary vein. An activating *EGFR*-mutation (L858R) had been detected in the tumor specimen. Osimertinib monotherapy had provided a dramatic radiographic response. The patient was diagnosed with potentially resectable disease after 4 months’ osimertinib treatment and was referred to our hospital. Complete resection with right lower lobectomy and combined resection of the left atrium was achieved. Pathological examination showed no viable tumor cells in the resected specimens. Postoperative course was uneventful. The patient is alive without tumor recurrence at 2 years after surgery.

**Conclusions:**

Pathological complete response was achieved with systemic treatment with osimertinib prior to surgery. Conversion surgery after osimertinib treatment may be safe and effective for NSCLC harboring activating *EGFR*-mutations.

## Background

Systemic treatment with a tyrosine-kinase inhibitor (TKI) of the epidermal growth factor receptor (EGFR) has become a standard treatment of care for advanced non-small cell lung cancer (NSCLC) harboring activating mutations in the EGFR gene such as deletions in the exon 19 (del19) and a point mutation in the exon 21 (L858R) [1]. First-generation EGFR-TKIs such as gefitinib and erlotinib provide durable tumor shrinkage associated with significant survival benefit in the majority of *EGFR*-mutated NSCLC patients. However, first-generation EGFR-TKIs, which are reversible inhibitors of tyrosine kinase, may provide an inferior pathological response, as compared with the impressive radiographical response. In fact, a retrospective study of 29 patients who received salvage surgery following systemic treatment with first-generation or second-generation EGFR-TKIs for advanced lung adenocarcinoma showed that major pathological response was achieved in only 9 (31.0%) patients, and that pathological complete response (pCR) was not achieved in any patient [2]. In a neoadjuvant setting for resectable NSCLC patients, a systematic review of clinical trials using first-generation EGFR-TKIs showed that pCR was not documented in 68 patients with clinical stage IIIA disease with mediastinal nodal involvement (N2) [3].

Osimertinib is a third-generation EGFR-TKI showing reversible and potent inhibitory effects of tyrosine kinase. As a randomized controlled trial (FLAURA) showed that osimertinib provided a superior survival benefit over first-generation EGFR-TKIs for advanced *EGFR*-mutated NSCLC [4], osimertinib monotherapy is recommended as the first-line treatment [1]. Here, we present a case of conversion surgery in which pCR was achieved with osimertinib treatment for initially unresectable adenocarcinoma of the lung. The present case not only indicates the safety and feasibility of surgery following osimertinib treatment, but also provides pathological evidence of potent anti-tumor efficacy of osimertinib.

## Case presentation

**Fig. 1:**
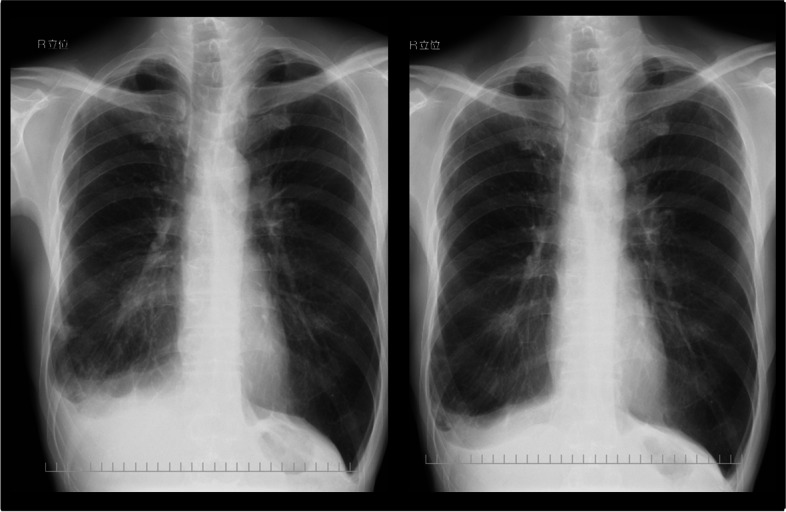
Chest roentgenogram before and after osimertinib treatment. Chest roentgenogram at the initial diagnosis showed a 3 cm mass in the right lower lung field (left). Chest roentgenogram after 4 months’ osimertinib treatment showed an apparent shrinkage of the mass (right)

**Fig. 2:**
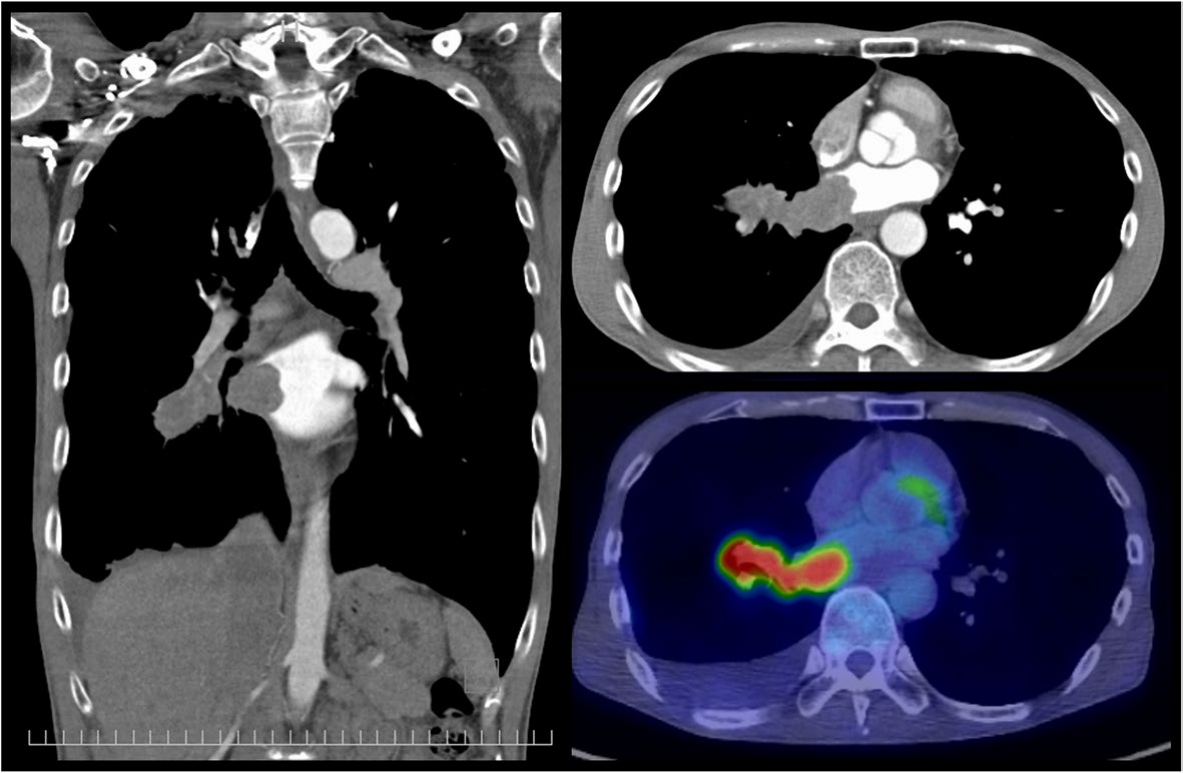
Computed tomography and positron emission tomography before osimertinib treatment. Computed tomography at the initial diagnosis (coronal section [left] and transverse section [right upper]) showed a 3.4-cm tumor originating from the right lower lobe and enlarged hilar nodes that invaded into the left atrium through the lower pulmonary vein. Positron emission tomography revealed a strong glucose uptake with the maximum standardized uptake value of 10.3 (right lower)

**Fig. 3:**
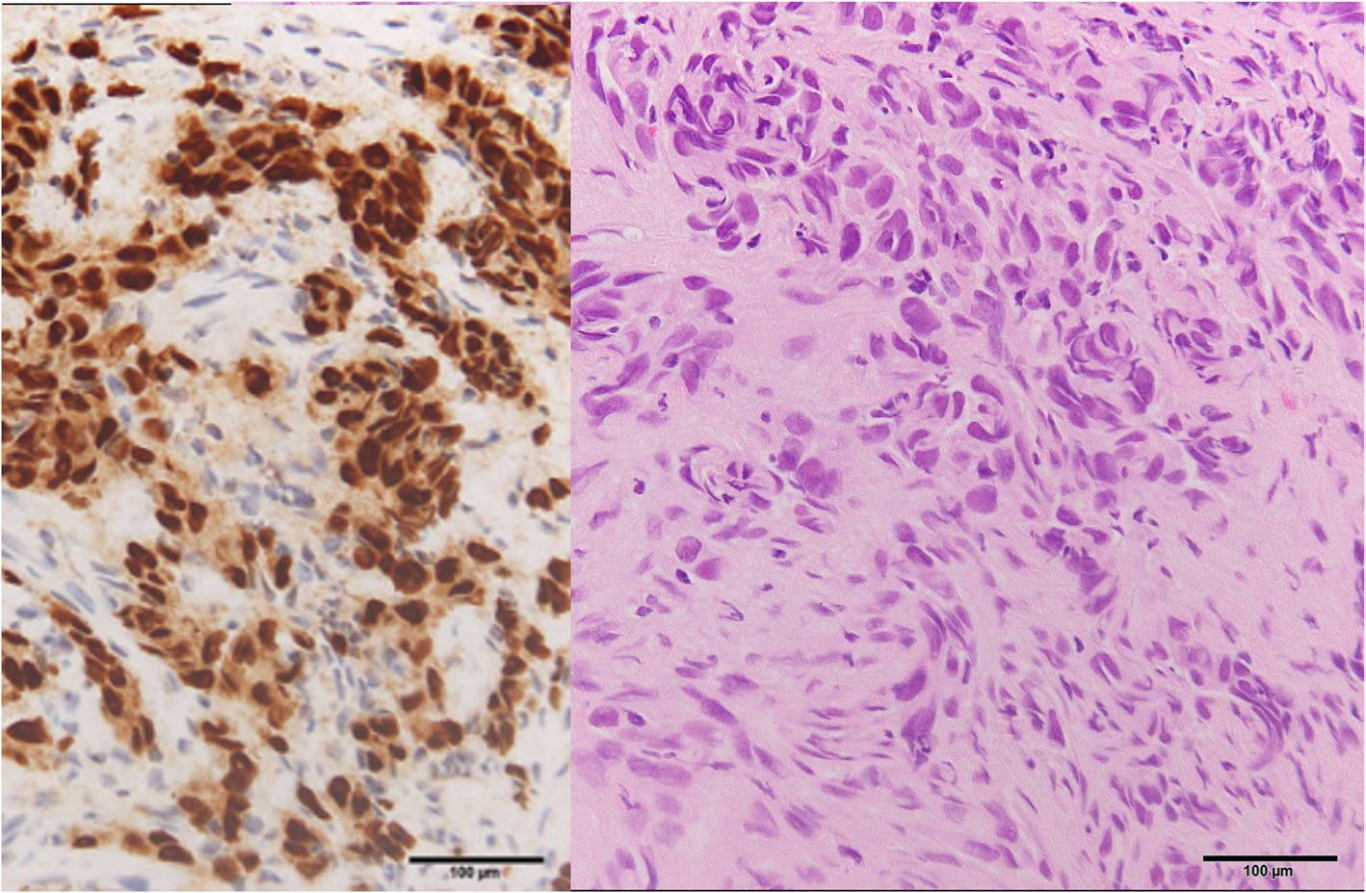
Pathological finding before osimertinib treatment. Pathological sections (hematoxylin and eosin staining [right]) obtained with transbronchial lung biopsy of the right lower lung tumor provided the diagnosis of poorly differentiated adenocarcinoma of the lung. Strong staining of thyroid transcription factor-1 was observed in the nucleus of tumor cells (left)

**Fig. 4:**
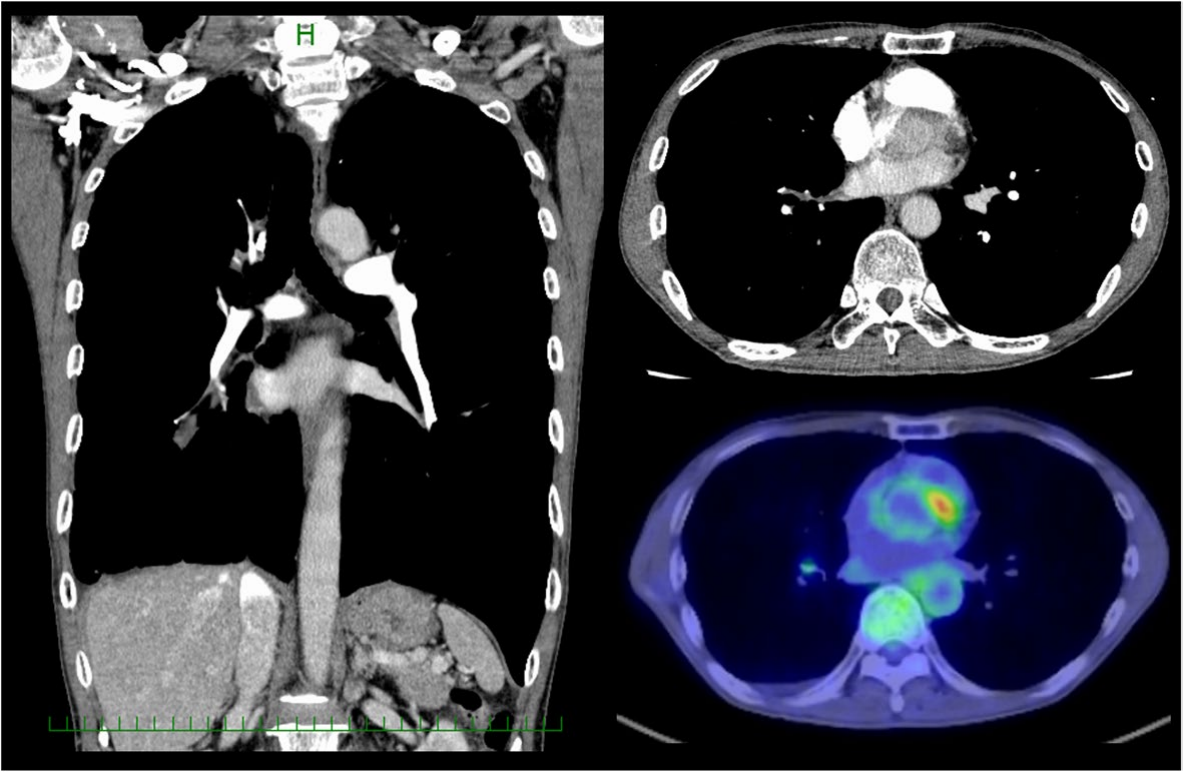
Computed tomography and positron emission tomography after osimertinib treatment. Computed tomography after 4 months’ osimertinib treatment (coronal section [left] and transverse section [right upper]) showed a marked tumor shrinkage. No apparent tumor invasion into the left antrum was observed. Positron emission tomography revealed no significant glucose uptake with the maximum standardized uptake value of 1.7 (right lower)

A 61-year-old Japanese man was admitted to our hospital for salvage surgery. Four months prior to the admission, he had been diagnosed with unresectable adenocarcinoma (clinical stage IIIA/T4N1M0; an activating *EGFR*-mutation [L858R]-positive; proportion of tumor expressing PD-L1, 50–74%) originating from the right lower lobe (Figs. 1, 2, and 3). Chest computed tomography (CT) had revealed a 3.4-cm tumor and enlarged hilar nodes that invaded into the left atrium through the lower pulmonary vein (Fig. 2). Definitive radiotherapy had not been indicated, as it may cause fatal bleeding due to rapid shrinkage of the tumor invading the left atrium. Osimertinib monotherapy (80 mg/day, daily) had been performed, which had provided a dramatic radiographic response (Fig. 4) with a 60% decrease in the sum of tumor diameters (3.4 cm [primary tumor] plus 3.6 cm [lymph nodes] before osimertinib treatment, and 2.3 cm plus 0.5 cm after osimertinib treatment; tumor response by RECIST criteria, partial response [PR]). As no distant metastasis had developed, the patient was diagnosed with potentially resectable disease and was referred to our hospital.

**Fig. 5:**
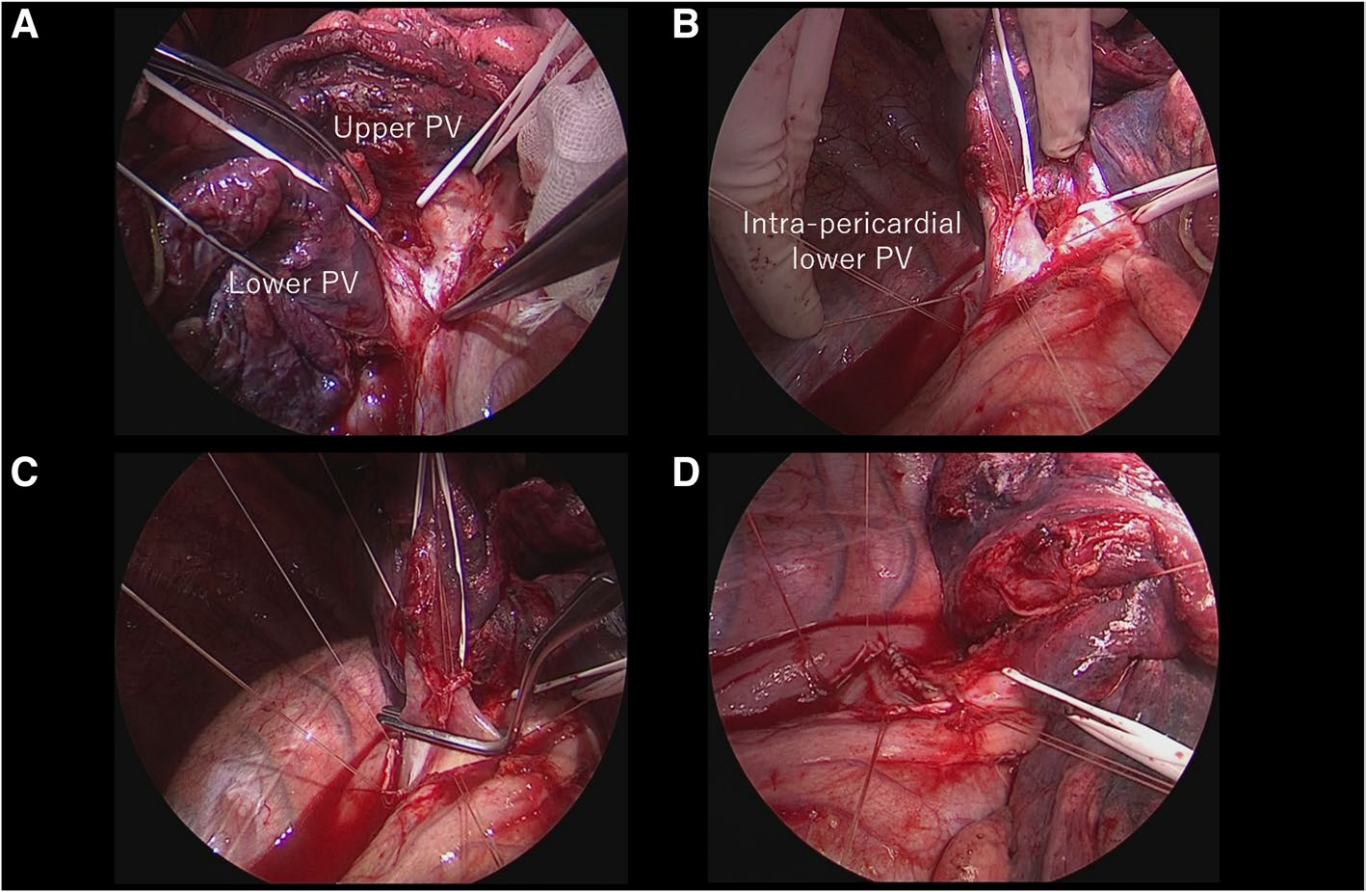
Intraoperative finding. No tumor invasion into the pericardium was observed (left upper, **A**). No tumor extent to the intrapericardial lower pulmonary vein (PV) or the left atrium was identified (right upper, **B**). The left atrium was safely clamped (left lower, **C**). The atrial cuff of the left atrium was resected, and the stump was closed with continuous 4–0 prolene suture (right lower, **D**)

Osimertinib treatment had been ceased for 7 days before surgery. As preoperative CT revealed no apparent residual tumor in the left atrium, achievement of complete tumor resection without cardio-pulmonary bypass was expected. Through an anterolateral thoracotomy, a right lower lobectomy with combined resection of the left atrium was performed. No pericardial involvement by the tumor was identified (Fig. 5A). The pericardial sac was opened, and the cytological examination of the pericardial effusion revealed no malignant cells. No apparent tumor extent to the intrapericardial lower pulmonary vein or the left atrium was identified (Fig. 5B). The left atrium was safely clamped without its elongation by dissection of the interatrial groove between the left atrium and the right atrium (Fig. 5C). The atrial cuff of the left atrium was resected. The stump of the left atrium was closed with a continuous 4–0 prolene suture (Fig. 5D). Frozen sections revealed negative surgical margins.

**Fig. 6:**
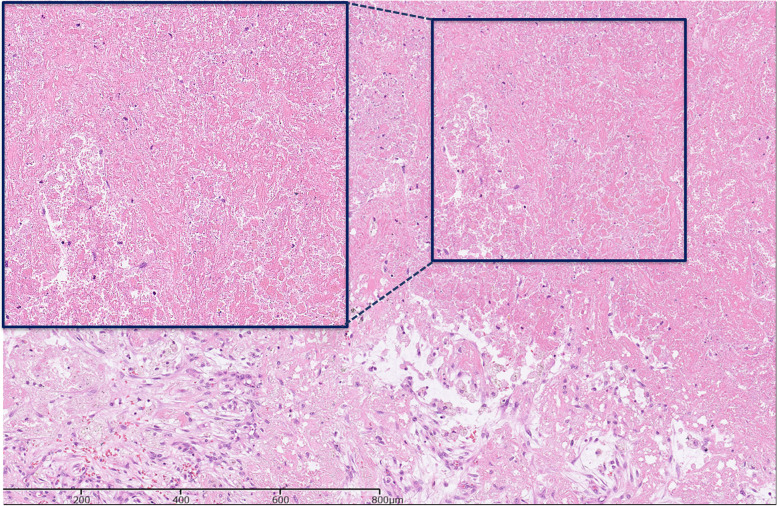
Pathological finding after osimertinib treatment. Pathological sections showed no viable tumor cells in any surgical specimen. Only extensive fibrosis with chronic inflammation was observed

Pathological sections showed no residual tumor (Fig. 6). Postoperative course was uneventful. Osimertinib monotherapy has been resumed after 3 months’ interruption after surgery. The patient is alive without tumor recurrence at 2 years after surgery.

## Discussion and conclusions

The present patient had been initially diagnosed with an unresectable disease by the previous physician due to tumor extent into the left atrium. Even when the length of the left atrial cuff was increased with dissection of the interatrial groove, the maximum extension was about 2 cm [5]. Accordingly, at the initial diagnosis, the involved left atrium had been too large to be safely clamped without cardiopulmonary bypass. After effective systemic treatment with osimertinib, the patient was diagnosed with resectable disease and was referred to our institute for conversion surgery.

Conversion surgery following systemic treatment using TKIs may be a feasible treatment option for selected patients with initially unresectable NSCLC harboring oncogenic gene alterations such as activating *EGFR*-mutations and rearrangements of the anaplastic lymphoma kinase (ALK) gene. In fact, a nationwide Japanese retrospective study of 36 patients who received salvage surgery including conversion surgery following EGFR-TKI (gefitinib, *n* = 26; erlotinib, *n* = 4; afatinib, *n* = 3) or ALK-TKI (crizotinib, *n* = 2; alectinib, *n* = 1) showed that grade 3 postoperative adverse events occurred only in 2 (5.6%) patients and that no death was reported within 90 days after surgery. The overall survival (OS) and recurrence-free survival (RFS) rates at 3 years after surgery were 75.1% and 22.2%, respectively [6]. The present case treated with osimertinib also indicated that conversion surgery following a third-generation EGFR-TKI is safe and feasible. In addition, for initially resectable NSCLC with oncogenic alterations, neoadjuvant treatment with TKIs may be effective [3]. An ongoing randomized clinical trial (NeoADAURA) may reveal whether neoadjuvant treatment with osimertinib is effective for resectable *EGFR*-mutated NSCLC [7].

A dramatic radiographic response to osimertinib treatment, as seen in the present case, has been commonly observed in *EGFR*-mutated NSCLC patients [4]. However, no case showing pathological evidence of pCR achieved with osimertinib has been reported. For advanced and previously-untreated *EGFR*-mutated NSCLC patients, the progression-free survival and overall OS were significantly longer in patients treated with osimertinib than in those treated with a first-generation EGFR-TKI [4]. For early resectable *EGFR*-mutated NSCLC patients, adjuvant treatment with a first-generation EGFR-TKI provided a modest improvement in RFS [7–10], and that with osimertinib provided a tremendous RFS benefit with a hazard ration of 0.17 [11]. Superior survival benefits of osimertinib observed in these clinical trials may indicate its potent anti-tumor effect, and the present case may provide direct pathological evidence for the superior anti-tumor efficacy of osimertinib.

The duration of preoperative and postoperative osimertinib interruption is controversial. Perioperative treatment with an EGFR-TKI may cause several postoperative adverse events mainly through inhibition of tyrosine kinase activity of normal cells, which may indicate a need of a long-term interruption such as 2–4 weeks’ cessation. On the other hand, interruption of treatment may cause tumor flaring, which can be avoided by a short-term interruption. Considering a milder toxicity profile of a third-generation EGFR-TKI, a shorter-time interruption of osimertinib treatment may be feasible. In fact, in a neoadjuvant trial with osimertinib (NeoADAURA) for NSCLC, it is recommended that surgery is performed just after the completion of preoperative treatment [7]. In the present case, we adopted a moderate-term interruption (7 days’ cessation) before surgery considering the advantages and disadvantages of treatment interruption.

The adequacy of adjuvant therapy, especially when pathological complete response (pCR) is achieved with preoperative treatment, remains to be discussed. Although pCR is a well-known surrogate indicator of a favorable prognosis in NSCLC patients who underwent surgery following neoadjuvant treatment, achievement of pCR does not warrant that no viable tumor cell is present in each patient (Ref. [12]). The prophylactic use of osimertinib following surgery may kill residual tumor cells, which can lead to a cure. On the other hand, adjuvant treatment may only cause adverse events in patients who actually harbor no viable cancer cells. I think that the choice to use osimertinib in the adjuvant setting comes down to each patient. In the present case, we discussed the necessity as well as the advantages and disadvantage of adjuvant osimertinib treatment with the patient. As a result, 3 months after surgery, osimertinib treatment has been resumed according to the patient’s wish.

In conclusion, we presented a case of *EGFR*-mutated lung adenocarcinoma with pCR in resected specimens after treatment with osimertinib. Conversion surgery after osimertinib treatment may be safe and effective for NSCLC harboring activating *EGFR*-mutations.

## Data Availability

The datasets used and/or analyzed during the current study are available from the corresponding author on reasonable request.

## Abbreviations

TKI: Tyrosine-kinase inhibitor
EGFR: Epidermal growth factor receptor
NSCLC: Non-small cell lung cancer
pCR: Pathological complete response
CT: Computed tomography
ALK: Anaplastic lymphoma kinase
OS: Overall survival
RFS: Recurrence-free survival
